# Estimating telomere length from whole genome sequence data

**DOI:** 10.1093/nar/gku181

**Published:** 2014-03-07

**Authors:** Zhihao Ding, Massimo Mangino, Abraham Aviv, UK10K Consortium, Tim Spector, Richard Durbin

**Affiliations:** 1 Genome Informatics, Wellcome Trust Sanger Institute, Hinxton, CB10 1SA, UK; 2 Department of Twin Research and Genetic Epidemiology, King's College London, London, WC2R 2LS, UK; 3 The Center for Human Development and Aging, New Jersey Medical School, Rutgers University, Newark, NJ 07103, USA

## Abstract

Telomeres play a key role in replicative ageing and undergo age-dependent attrition *in vivo*. Here, we report a novel method, TelSeq, to measure average telomere length from whole genome or exome shotgun sequence data. In 260 leukocyte samples, we show that TelSeq results correlate with Southern blot measurements of the mean length of terminal restriction fragments (mTRFs) and display age-dependent attrition comparably well as mTRFs.

## INTRODUCTION

Telomeres cap the ends of chromosomes and are critical for the maintenance of genome integrity. In humans, telomeres comprise sequences of 5–15 kb TTAGGG tandem repeats and their telomere binding proteins (1). In the absence of telomerase or the alternate pathway, telomeres undergo progressive attrition, which ultimately leads to replicative senescence or apoptosis. Thus, telomere length is an indicator of replicative history and replicative potential—two features of great importance to human health and disease (2).

Standard methods for telomere length measurement are generally classified into three categories: (i) Southern blot analysis of the terminal restriction fragments that measures the average length (mTRF) and length distribution of telomeres in a sample of cells (3); (ii) methods that examine variation in telomere length between chromosomes and cells, i.e. fluorescence *in situ* hybridization (FISH) techniques, including Q-FISH (4) and Flow-FISH (5); and (iii) quantitative polymerase chain reaction (qPCR)-based techniques that measure telomere deoxyribonucleic acid (DNA) content in relative units (compared to single gene DNA) (6).

Next-generation sequencing has now provided an opportunity to obtain genomic information computationally. Shotgun sequence data contains sequencing reads from the telomeres just as any other region of the genome. However, little information about the telomeres can be gained from standard alignments of these reads to the reference sequence. This is because the repetitive nature of the telomeric regions means that it is not possible to assign with confidence the exact origins of the reads, and also since in the human reference sequence (build GRCh37), the ends of most chromosomes are simply stretches of Ns, representing unknown nucleotides.

Instead, previous studies (7) have shown that information on telomere length is contained in the number of telomere motif copies (TTAGGG or CCCTAA) found in reads. Parker *et al.* (8) applied this idea to cancer samples. However, cancer samples typically suffer from aneuploidy, complicating the validation of their results by method such as qPCR (it relies on normalising against a unit copy region). This may be the reason why the measures in (8) only converge to a low resolution telomere status, defined as either gain, no change or loss relative to normal control. Additionally, the vast majority of the samples were paediatric with mean age 7.5 years, and they did not demonstrate a relationship between age and their sequence-based telomere length measurement.

Here, we further examine the relationship between reads containing telomere repeat sequence and telomere length, and describe software for estimating telomere length based on genome-wide sequence data. We demonstrate our method on 260 leukocyte samples (aged 27–74 years, mean age 51 years) from the TwinsUK cohort (9) that have both Illumina 100 bp paired-end whole genome sequence and telomere length measurements using Southern blot mTRFs. We also investigate 96 samples from the 1000 Genomes Project (10) that have both whole genome and exome data.

## MATERIALS AND METHODS

**Figure 1.**
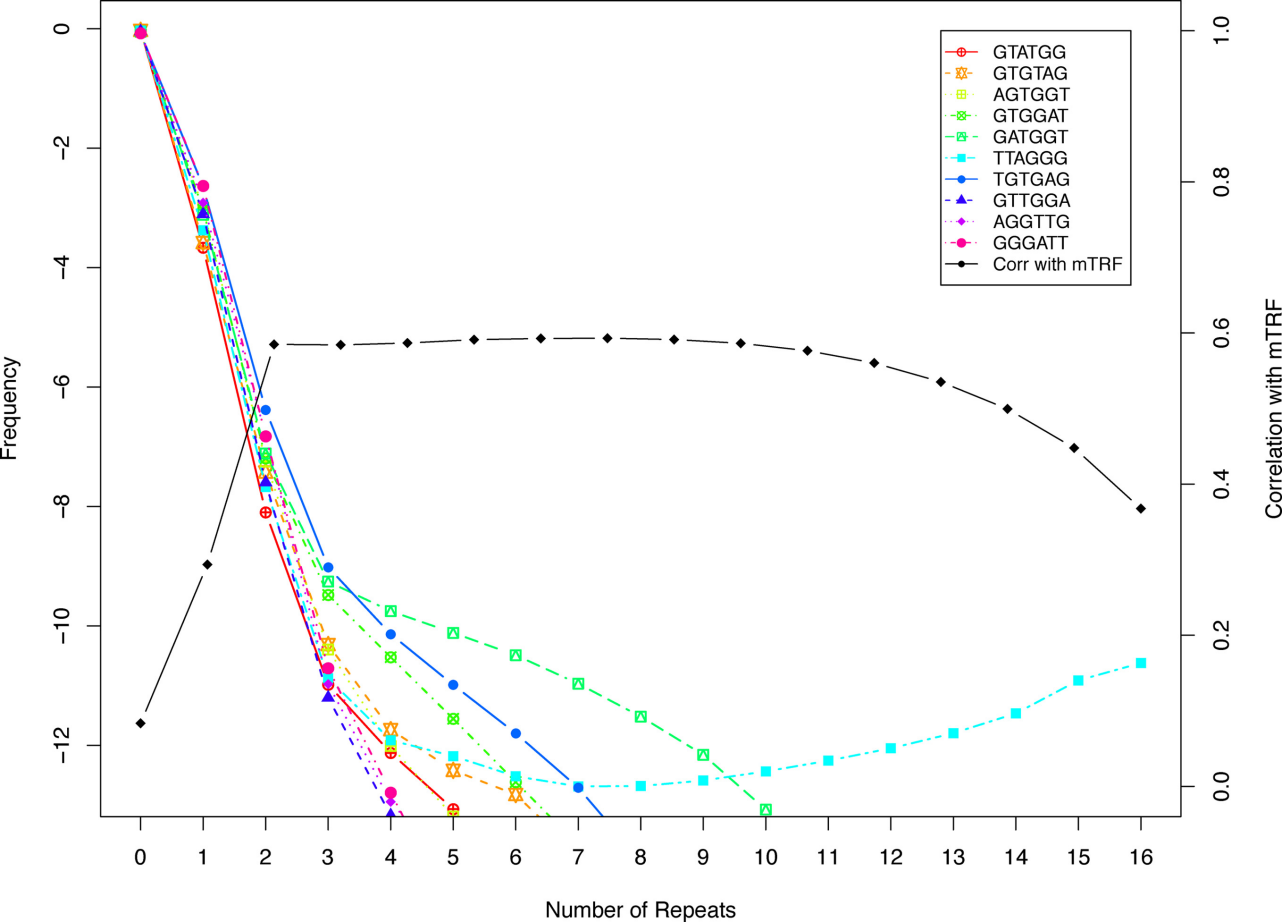
Identification of telomeric reads. In cyan, the log scale frequencies of reads with different numbers of TTAGGG repeats averaged across the 260 TwinsUK samples, with corresponding plots for permutations of TTAGGG in other colours. In black, the correlation of TelSeq to mTRF as a function of the threshold *k* for the number of repeats per read used in the TelSeq measurement.

We first examined the frequency of reads from the TwinUK dataset with different numbers of copies of TTAGGG and also each non-cyclical permutation of TTAGGG as a control. The frequencies of all non-TTAGGG hexamers showed a monotonic decay as the number of repeat units increased, with none occurring in a read more than 11 times (Figure 1). In contrast, beyond seven repeats there was an increase in the number of reads containing TTAGGG. We defined reads as telomeric if they contained *k* or more TTAGGG repeats, with a default threshold value of *k* = 7. These can then be translated into an estimate of the physical length via a size factor *s* and a constant length *c* in *l = t_k_sc*, where *l* is the length estimate, *t_k_* is the abundance of telomeric reads at threshold *k* and *c* is a constant for genome length divided by number of telomere ends 46 (23 × 2). The total number of reads could be a good measure of sequence depth and thus a reasonable choice for *s*. However, studies have shown that DNA molecules in a sequencing library are not sampled and sequenced with equal probability, but instead are subject to biases due to different molecular properties such as GC composition—a high value of which favours more amplification in the PCR step (11). This results in different representations of genome regions and makes defining *s* as the total read number not a good estimate. Instead, we define *s* as a fraction of all reads with gas chromatography (GC) composition between 48 and 52%. The range was chosen to be close to the telomeric GC composition, which is 50% at the TTAGGG dense regions (see Supplementary Figure S1 for results for other GC composition ranges). This fraction is then converted to a mean telomere length estimate in kilobases by multiplying by the cumulative length of genomic regions with the same GC composition *c*. Considering the GC composition removed an important source of experimental error; and effectively increased the signal by nearly 2-fold, as measured by the correlation between experimental estimates (Supplementary Figure S1). This method is implemented in a program TelSeq which reads one or more Binary Alignment/Map (BAM) files files and returns a report with one row per read group present in the input.

We employed simulated datasets to investigate the effect of sequencing coverage. This was also to discover the minimum amount of sequence required for reasonable length estimation. We chose the reference sequence (GRCh37) of human chromosome 1 as the sequence source, but with 30 kb nucleotides (including unknown nucleotide Ns) removed from each end and replaced with telomere repeat sequences of the same length. We then simulated 255 synthetic BAMs using the software SimSeq (https://github.com/jstjohn/SimSeq) with sequencing coverage in individual BAMs varying from 0.2*X* to 10*X* in 0.2*X* increments (Supplementary Methods, Supplementary Figure S2). When applied to all BAMs, TelSeq predicted telomere length to be on average 29.4 kb with 1.47 kb standard deviation (SD) (5% of mean). Significant higher variation was seen when coverage was below 2.5*X* (*F* = 10.5, *P* = 2.2E−16 in *F* test) when compared to results from the higher coverage BAMs (Supplementary Figure S2). For BAMs with >2.5*X* coverage, TelSeq predicted telomere length to be 29.5 kb with 0.71 kb SD (2.4% of mean).

## RESULTS AND DISCUSSION

**Figure 2.**
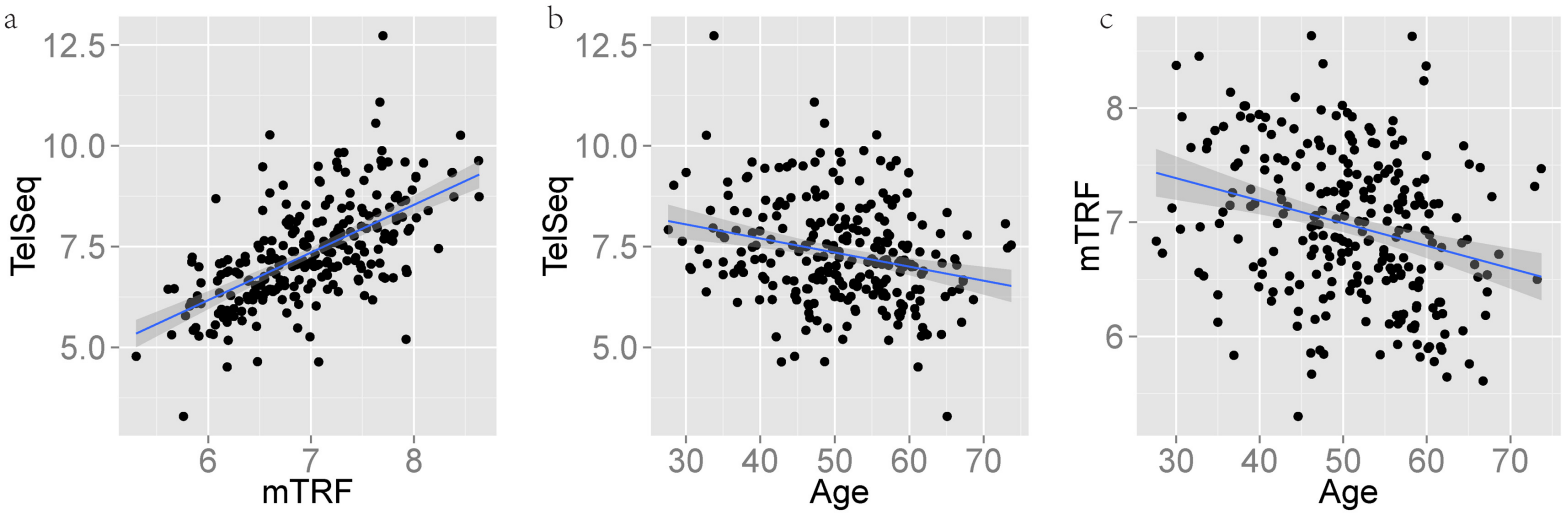
Comparison of TelSeq with experimental measure and age in TwinsUK samples. (a) TelSeq estimate of average telomere length plotted against mTRF estimate; TelSeq (b) and mTRF (c) estimates plotted against age. All average length estimates in kilobases and ages in years.

When TelSeq was applied to the TwinsUK data, the estimates of leukocyte telomere length (LTL) correlated well with the mTRFs measurements across a range of choices of *k*, with correlation *ρ* = 0.60 at the default threshold *k* = 7 (*P* < 10E−16; Figure 2a). We next examined the relationship between the TelSeq-based LTLs and age of the donors. Given the wide inter-individual variation in LTLs for persons of the same age and the impact of environmental factors on this parameter, the correlation between LTL measurements and age in cross-sectional studies, including TwinsUK, is usually modest (12,13). Nevertheless, since the relationship between measurement and donor age depends on the true LTL value, the correlation provides a means for independent assessment of the informativeness of different experimental techniques for estimating LTL. The TelSeq measurement displayed correlation of *ρ* = −0.24 (explaining 6.5% variance of age, Figure 2b) with age, comparable to that of mTRF (Figure 2c; *ρ* = −0.26, explaining 7.5% variance of age) (Supplementary Method). The difference between −0.24 and −0.26 is not significant in a *t*-test using a SD derived by bootstrapping (*P* = 0.79, Supplementary Methods, Supplementary Figure S4). The coefficient of multiple correlation between age and both LTL and mTRF was higher than either individual correlation (*ρ* = −0.34, explaining 9% variance of age); both measurements contributed significantly to the underlying linear regression model (*P* = 0.016, *t*-test for the TelSeq term; *P* = 0.009, *t*-test for the mTRF term, Supplementary Methods). This implies that neither TelSeq nor mTRF captured all the information available, and that TelSeq contains additional information independent from that provided by mTRF.

**Figure 3.**
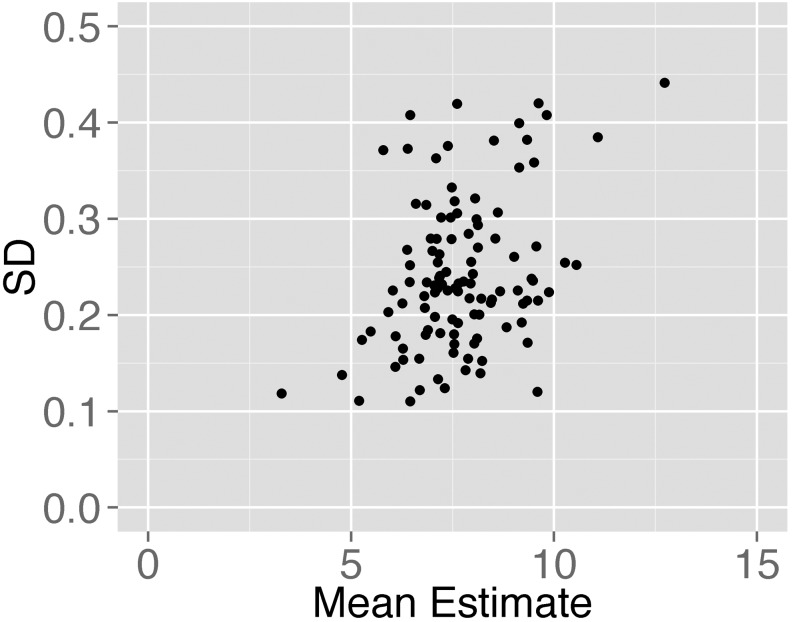
Sequencing lane variation in TelSeq measures. For each sample that was sequenced on more than 10 lanes, the SD of the length estimates across lanes is plotted against the mean length estimate. The CV, defined as the ratio of the SD to the mean, varies between 1.3 and 6.4%, with mean 3.17% and SD 0.98%.

A subset of our samples was sequenced on multiple lanes in separate runs. They can be considered as technical replicates and used to assess the variability of TelSeq measures. The coefficient of variation (CV) was computed as the ratio of the SD to the mean across the technical replicates for each sample. We selected 110 samples that were sequenced on more than 10 lanes to evaluate the CV and observed an average value of 3.17% with 0.98% SD (Figure 3), comparable to or smaller than that from the experimental measurements (14).

Notably, the TelSeq estimate of telomere length was consistently shorter than the mTRF estimate (mean 5.63 kb compared to 6.97 kb), and the mean rate of shortening per year was consistently greater (34.5 bp/year against 19.8 bp/year) (Figure 2b and c). The mTRF measurements reflect the average distance from a restriction enzyme site (HinfI/RsaI or HphI/MnlI) to the end of a chromosome, and hence overestimate the canonical region of the telomeres of TTAGGG repeats only. Kimura *et al.* (14) obtained a similar figure of around 1 kb for the additional sub-telomeric length included in an mTRF measurement. The difference between the TelSeq and mTRF estimates changes as the TelSeq threshold *k* changes, reflecting inclusion of different amounts of subtelomeric sequence (Supplementary Figure S5); although the correlation between TelSeq and mTRF remains similar across a range of values of *k* (Figure 1).

In addition to whole genome sequence data, a large number of samples have exome sequence data collected by enrichment of whole genome shotgun sequencing libraries using capture reagents. In theory, if the exome capture works perfectly, it would not be possible to use these data for our method. However, in practice with current technology, a typical exome sequencing output contains some fraction (typically 10–50%) of sequence that is off-target, i.e. not exonic. This fraction represents information on the rest of the genome and can be used to estimate relative telomere length by our method. To test this approach, we selected 96 samples from the 1000 Genomes Project pilot that have matched whole genome and exome sequence and applied TelSeq to both datasets. We found that when we classify telomeric reads as those containing more than three TTAGGG hexamers, estimates of telomere length from the two datasets started to be tightly correlated (Supplementary Figure S6). Using our default threshold of *k* = 7, the two measures have a Spearman Rank correlation coefficient 0.78. This result suggests that TelSeq can effectively work with exome data, which substantially extends its potential applications.

In conclusion, we have demonstrated an approach for measuring telomere length using whole genome or exome sequencing data. This is the first study to our knowledge to evaluate in detail the relationship between the frequency of telomere repeats and telomere length; and also to validate extensively with experimental measurements in a representative large sample cohort with a wide range of ages. We have implemented our methods in a software package that has been made freely available under the GPL open software licence (https://github.com/zd1/telseq). This allows any cohort with existing genomewide sequence data, including increasingly many cancer genomics and epidemiological cohort studies, to produce a validated measure of telomere length at effectively no cost, with no need for the further sample collection and experimental procedures required by other methods of ascertaining telomere length.

## SUPPLEMENTARY DATA

Supplementary Data are available at NAR online, including Supplementary References (15).

SUPPLEMENTARY DATA
