# Supplementary material for: Estimating telomere length from whole genome sequence data
Source: Nucleic Acids Res. 2014 Mar 7;42(9):e75. doi: 10.1093/nar/gku181 (PMC4027178; doi:10.1093/nar/gku181)
Supplement: SUPPLEMENTARY DATA [file supp_42_9_e75__index.html]

Estimating telomere length from whole genome sequence data — SUPPLEMENTARY DATA 

# Estimating telomere length from whole genome sequence data

## SUPPLEMENTARY DATA

**Files in this Data Supplement:**

- SUPPLEMENTARY DATA
