# Supplementary material for: Estimating telomere length from whole genome sequence data
Source: Nucleic Acids Res. 2014 Mar 7;42(9):e75. doi: 10.1093/nar/gku181 (PMC4027178; doi:10.1093/nar/gku181)
Supplement: SUPPLEMENTARY DATA [file supp_gku181_nar-03708-met-k-2013-File002.pdf]

Supplementary Information

Estimating telomere length from whole genome sequence data

Zhihao Ding[1], Massimo Mangino[2], Abraham Aviv[3], UK10K Consortium, Tim Spector[2], Richard Durbin[1]*

[1] Genome Informatics, Wellcome Trust Sanger Institute, Hinxton, CB10 1SA, UK
[2] Department of Twin Research and Genetic Epidemiology, King's College London, London, WC2R 2LS, UK
[3] The Center for Human Development and Aging, New Jersey Medical School, Rutgers University, Newark, NJ 07103, USA

METHODS

Samples and sequencing data.

The 260 UK10K individuals investigated in this study were all female aged 27 -74 years (mean age 51 years) from the TwinsUK cohort(1) (http://www.twinsuk.ac.uk/), except for 5 pairs of dizygous twins, the rest were all unrelated. Leukocyte telomere lengths of these individuals as mTRFs were measured using Southern blot. Whole genome sequencing was conducted using the Illumina HiSeq technology, yielding sequencing reads with coverage ranging from 4X to 16.6X (average 6.5X, pooled across lanes). Most samples were sequenced on multiple lanes (median=4 lanes, median per lane coverage=1.54X). These can be considered as technical replicates. For the generality of the method, as some studie may not have any technical replicates, we merged all lanes before analysis. However, when lanes analysed separately and the telomere length estimate calculated as the mean across lanes, weighted by lane yield, the sampling error was further reduced and the correlation with mTRF was stronger(ρ=0.62 with mTRF as opposed to ρ=0.60 when merged). Twelve individuals with a much higher duplication rate (more than 3 fold that of other samples) were investigated for duplication effect but excluded from the rest of the analysis (Supplementary Fig 3).

Sequence data are available from the European Genome-phenome Archive (EGA) study number EGAS00001000108, submitted by UK10K (http://www.uk10k.org). 1000 Genomes Project sequence data were downloaded from http://www.1000genomes.org.

Normalization and length measurement.

The TelSeq telomere length estimate in kilobase pairs is given by $l=t_k s c_g$, where l is the length estimate, t_k is the abundance of telomeric reads at threshold k and c_g is a constant for the cumulative length of genome sequences with GC composition g, divided by 46 (the number of telomere ends, 23×2). To calculate g we divide the reference sequence into 100bp consecutive bins and add 100bp to c_g if the GC composition of the bin is within g. Here g is chosen to be [48%, 52%], close to the telomeric GC composition, which is 50% at the TTAGGG dense regions. Normalising only with reads close to 50% GC composition avoids bias due to uneven GC in sequencing library representation(2) and improves signal substantially (Supplementary Fig1).

Simulation.

SimSeq (https://github.com/jstjohn/SimSeq) was used for simulating Illumina pair-end reads. Human chromosome 1 (GRCh37) was used as the sequence source. 30,000 nucleotides of sequence, including strings of Ns that are placeholders for unknown nucleotides at the ends, were removed from each end, and the same of length of TTAGGG repeats were appended instead. This generates a new

chromosome sequence of same length but with known telomere length (30kb). We simulated reads using parameters: -1 100 -2 100 --insert_size 500 --insert_stdev 200, with coverage ranging from 0.2X (498,501 reads) to 10X (24,925,063 reads) in increments of 0.2X greater depth and with duplication rate fixed at 5%. For each setting we repeated the simulation 5 times. In total 255 BAMs were simulated. We then used TelSeq to estimate telomere length (Supplementary Fig 2).

Associations

The Pearson's Correlation Coefficient was calculated using the "*cor*" function of the R language (http://www.r-project.org/). The regression between age and TelSeq and between age and mTRF was calculated using the "*lm*" function of R in models *lm*(*age ~ telseq*) and *lm*(*age ~ mTRF*). Two measures were also included in one model *lm*(*age~telseq + mTRF*) as two independent fixed effects. A t-test was done for each of the two regression coefficient (beta) against null hypothesis beta=0, the results of which can be seen in the output of the *summary* function.

Calculating variance explained

To compute the proportion of variance of age explained by mTRF, we used the "*cor*" function in R *cor*(*age, mTRF*, method="pearson")^2. And the same was done for TelSeq. To compute the additional variance that can be explained by mTRF while controlling for TelSeq, we firstly obtained the residuals from a regression between age and TelSeq (*x* <- *lm*(*age~TelSeq*)*$residuals*); and then used the residuals to compute the additional variation explained (cor(*x,mTRF*)^2). The same procedure was done for TelSeq.

Comparing the correlation coefficients with age by the two methods

To test whether the difference is significant in the strength of associations between age and each of two measures – ρ = -0.24 for TelSeq and ρ = -0.26 for mTRF, we conducted bootstrapping using R (*sample(sample_index,sample_size,replace=TRUE)*)) sampling our cohort 1000 times, from which we obtained an estimate for the standard deviation of ρ for mTRF (0.052) and TelSeq(0.056). We can then compute the Student-t statistic $t = (\rho_{telseq} - \rho_{mTRF})/sqrt(sd^2_{TelSeq} + sd^2_{mTRF})$ for hypothesis testing (Supplementary Fig 4).

FIGURES

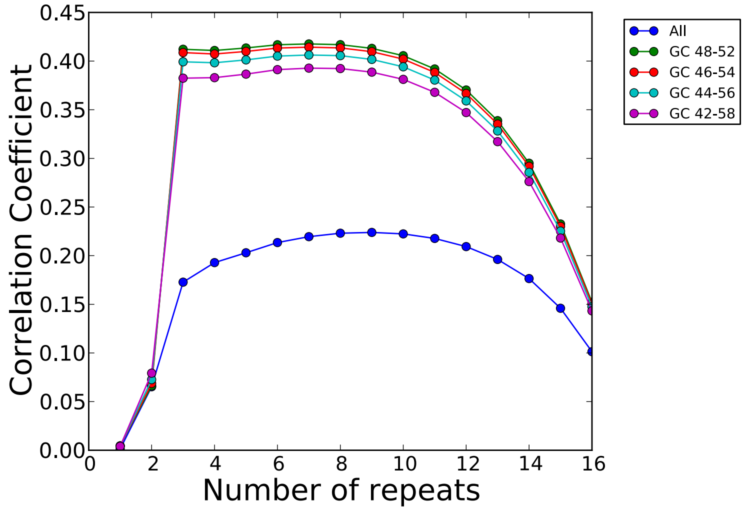

Supplementary Figure 1. Normalising by reads with similar GC improves the performance of TelSeq. It is known that read abundance in a sequencing library is affected by the GC composition of a read, a bias primarily introduced in the PCR step where high GC reads get amplified more often due to their high molecular affinity. Thus, using reads with similar GC content as background accounts for this molecular property and reflects the signal to noise ratio more accurately. To demonstrate this we evaluated the performance of TelSeq, as measured by the correlation with mTRF, when normalised by reads from different GC groups, 42%-58% (purple), 44%-56% (light green), 46%-54% (red), 48%-52% (dark green) as well as by all reads (blue). The result showed that there was a gradient increase to the correlation when GC range approaches 50%. And in all these cases, the correlation was much higher than that when all reads were used from a library. Here the analysis was done for the whole range of threshold *k*, the number of TTAGGG repeats in a read.

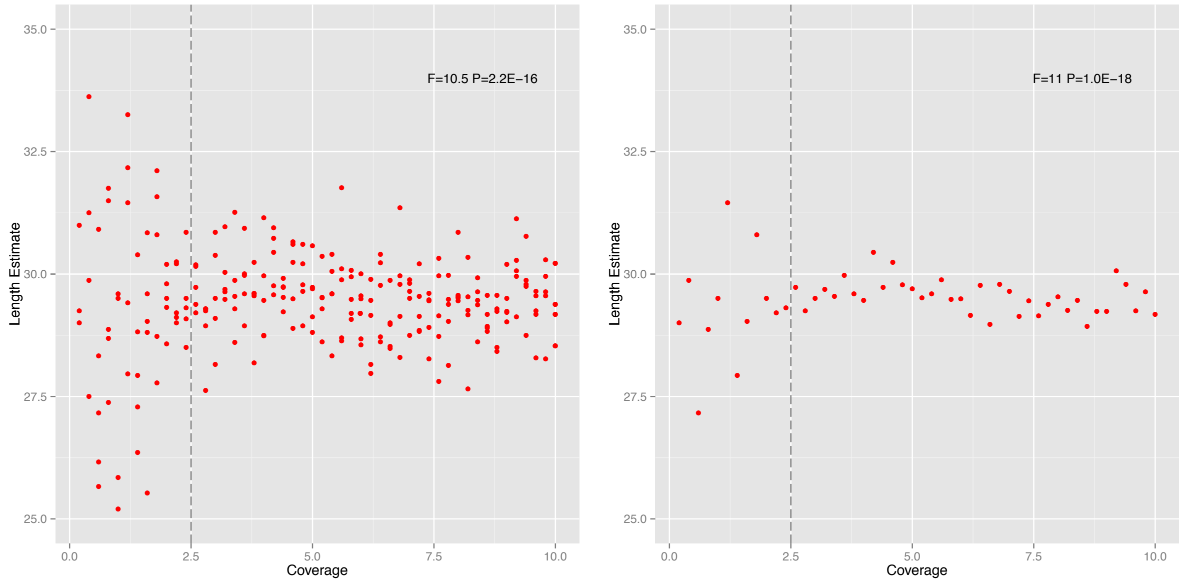

Supplementary Figure 2. The effect of sequencing coverage on TelSeq measurement, assessed by simulation. A group of BAMs were simulated using software SimSeq (https://github.com/jstjohn/SimSeq) (Supplementary Method). Sampling noise is substantially higher when the coverage is below 2.5X (mean=29.4kb, variation=5% of mean), compared to when coverage is above 2.5X (mean=29.5kb, variation=2.4% of mean) (left hand plot). The mean estimates are close to the true value 30kb independent of coverage. When using the weighted average of 5 BAMs for each coverage group (right hand plot), the variation is much smaller (1% of mean). This is justified theoretically by the relationship X~N(μ,σ), mean(X)~N(μ, σ/sqrt(n)), where *n* is the sample size. The coefficient of variation across lanes per sample is on average 3.2% (main Figure 3).

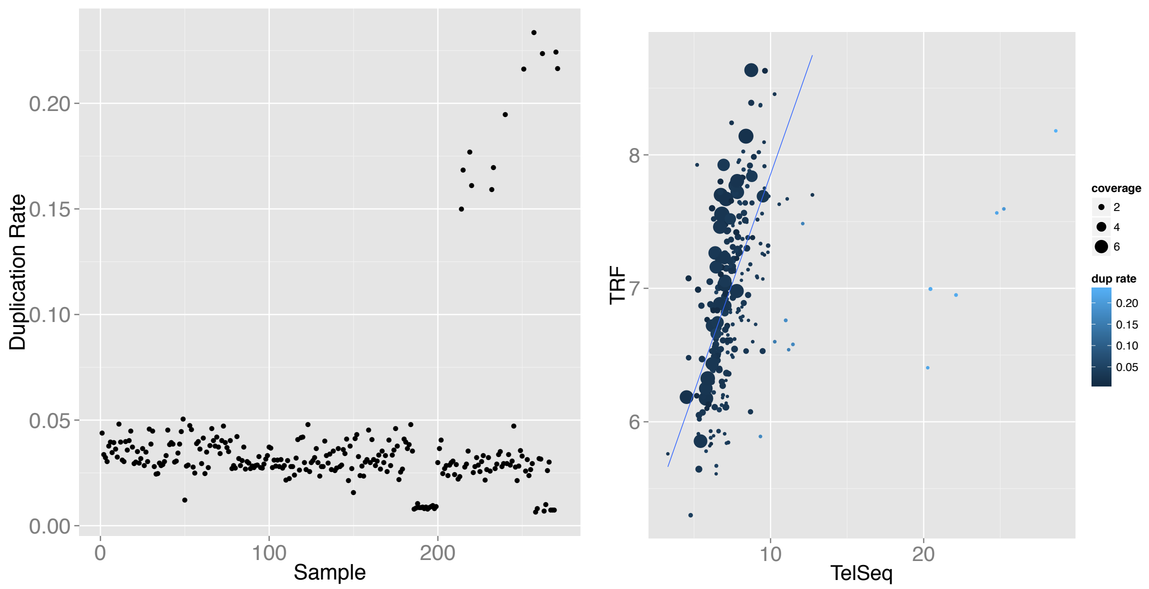

Supplementary Figure 3. The effect of duplication rate and coverage to TelSeq performance. In essence, TelSeq relies on sampling of genomic regions from a sequencing library. Coverage and duplication thus affect the translation of a relative measure into an absolute one. Low coverage indicates insufficient sampling and thus results in high variation in estimation (Supplementary Figure 2) while high duplication suggests over enrichment of certain genomic regions and thus changes the translation factor *c* (Supplementary Method). In whole genome sequencing high duplication rate indicates low library complexity and loss of information. Twelve of our samples were found to have an exceptionally high duplication rate (>3 fold greater than the rest, left panel), and were outliers when regressing against mTRF (right panel). We based our evaluation on samples with duplication rate below 10%, which is typically what is expected for whole genome sequencing.

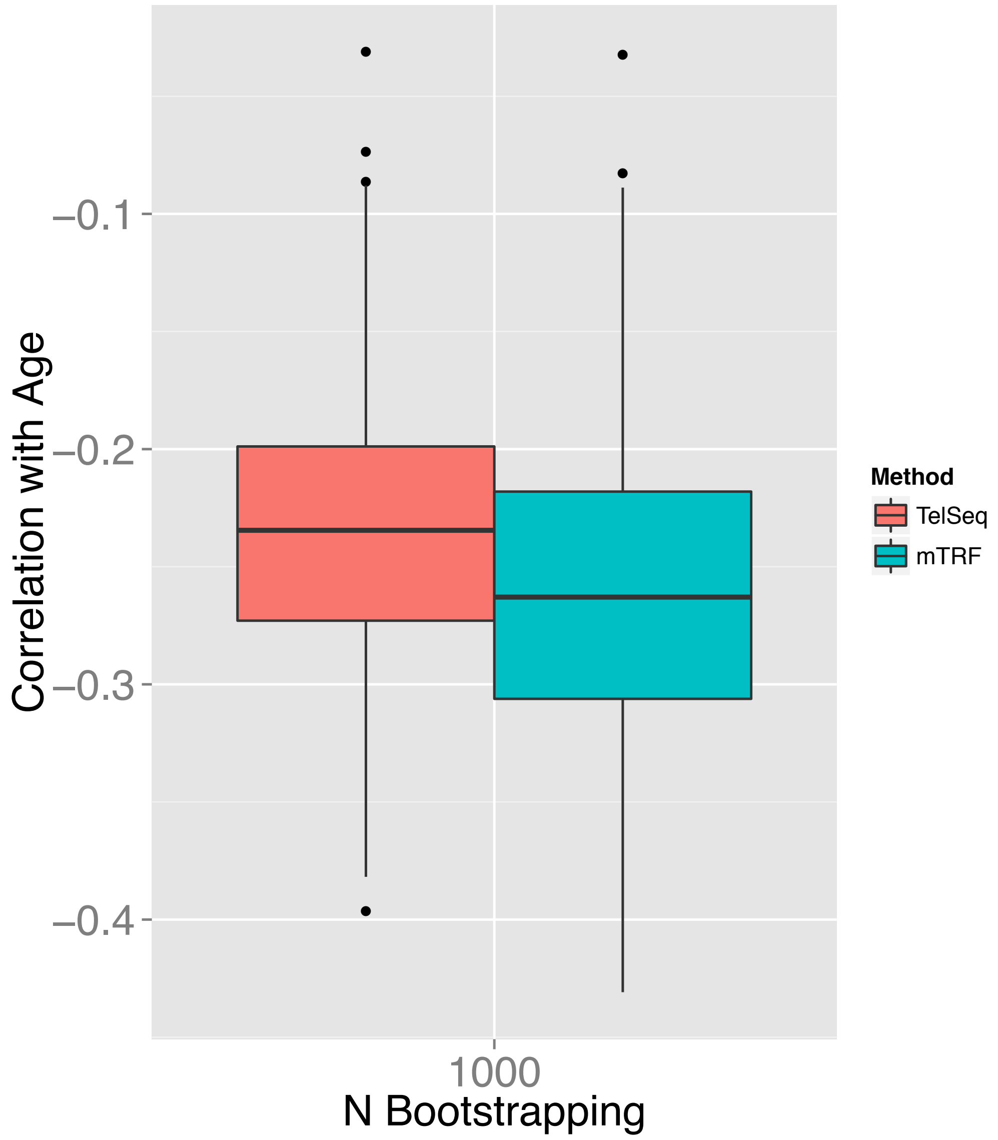

Supplementary Figure 4. Compare correlation coefficient obtained from mTRF and TelSeq. To compare the correlation coefficients between age and telomere length estimates from TelSeq and mTRF, we conducted 1000 bootstraps with replacement from the data set to obtain an estimate of the standard deviations of the correlation estimates ρ. We can then perform a t-test for whether the difference between the observed values -0.24 and -0.26 is significant. The result gave t=0.26, p=0.79, which suggest no statistical difference between the coefficients obtained from the two measurements.

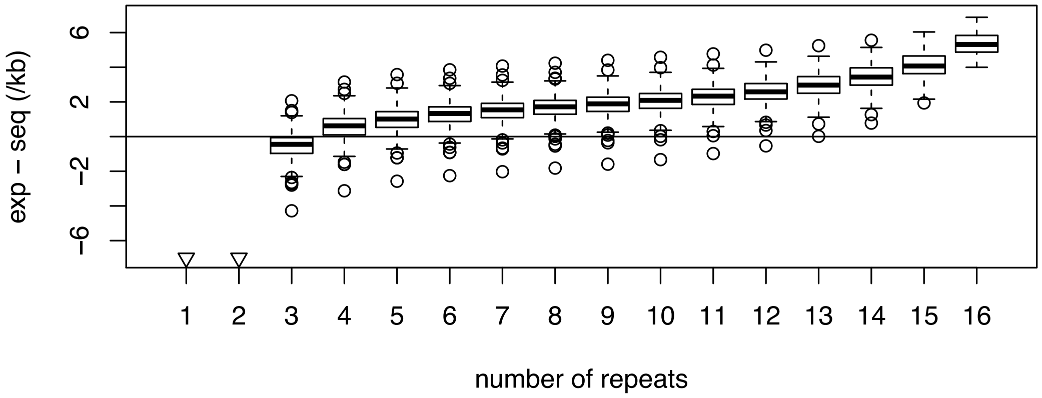

Supplementary Figure 5. The mTRF measurement is longer than TelSeq estimates across a range of values for the choices of TelSeq threshold (*k*). The difference between mTRF and TelSeq is 1.49kb at *k*=7, and 5.34kb at *k*=16. The difference reflects the fact that mTRF measures the average distance from subtelomeic regions, where the excision sites of restriction enzymes exist, to the chromosome ends, while TelSeq approaches include only the ends when choosing a large *k*.

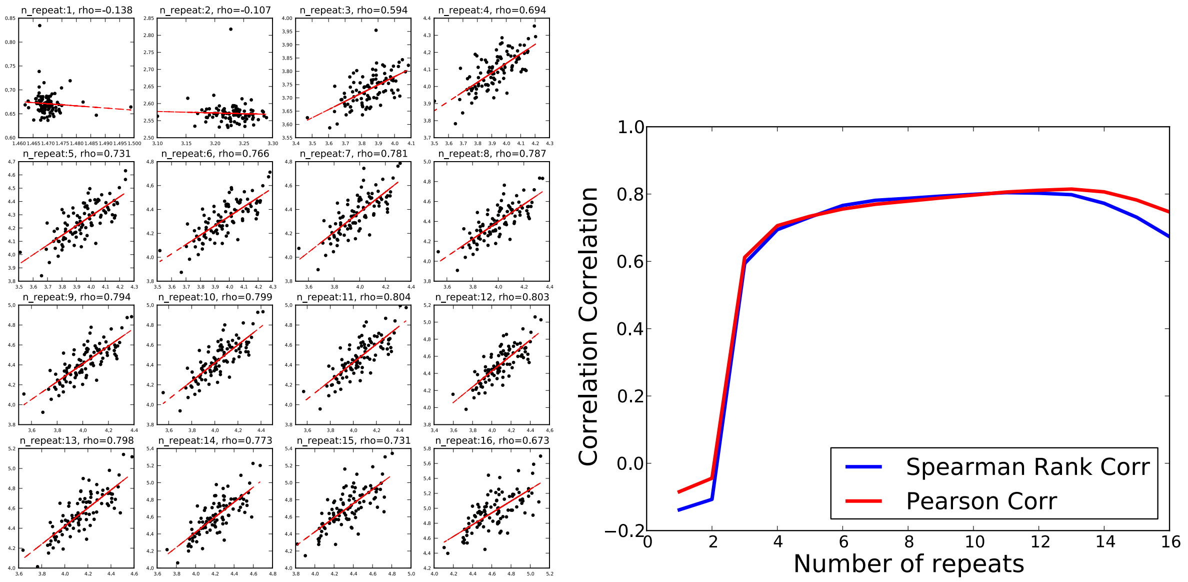

Supplementary Figure 6. TelSeq estimates from exome data are highly correlated with those from whole genome data in 96 samples from the 1000 Genomes Project with matched whole genome sequences and exome sequence data. (left) Scatter plots for TelSeq estimates from matched whole genome sequence and exome sequence at different thresholds of *k*, the amount of TTAGGG repeats in a read. Panels are organised from left to right, top to bottom as *k* increases from 1 to 16, where in each plot X axis is the estimates from the whole genome sequences and y axis is the estimates for the matched exome sequences. A correlation coefficient is calculated for each panel and plotted in right panel. The two measurements start becoming tightly correlated with each other when *k*>= 3.

REFERENCES:

1. Moayyeri, A., Hammond, C.J., Valdes, A.M. and Spector, T.D. (2013) Cohort Profile: TwinsUK and healthy ageing twin study. *International journal of epidemiology*, **42**, 76-85.
2. Dohm, J.C., Lottaz, C., Borodina, T. and Himmelbauer, H. (2008) Substantial biases in ultra-short read data sets from high-throughput DNA sequencing. *Nucleic acids research*, **36**, e105.
3. Abyzov, A., Urban, A., Snyder, M. and Gerstein, M. (2011) CNVnator: an approach to discover, genotype, and characterize typical and atypical CNVs from family and population genome sequencing. *Genome research*, 974-984.
